# Estimation of the Ultraviolet-C Doses from Mercury Lamps and Light-Emitting Diodes Required to Disinfect Surfaces

**DOI:** 10.6028/jres.vol.126.025

**Published:** 2021-08-20

**Authors:** Pablo Fredes, Ulrich Raff, Ernesto Gramsch, Marcelo Tarkowski

**Affiliations:** 1 Hydraluvx, Minerva 2576, Maipú, Santiago 9254013, Chile; 2 Physics Department, University of Santiago of Chile, Av. Ecuador 3493, Estación Central, Santiago 9170124, Chile

**Keywords:** disinfection, dose, dose distribution, light-emitting diodes, modeling, simulation, surface disinfection, ultraviolet-C

## Abstract

Disinfection of surfaces by ultraviolet-C (UV-C) radiation is gaining importance in diverse applications. However, there is generally no accepted computational procedure to determine the minimum irradiation times and UV-C doses required for reliable and secure disinfection of surfaces. UV-C dose distributions must be comparable for devices presently on the market and future ones, as well as for the diverse surfaces of objects to be disinfected. A mathematical model is presented to estimate irradiance distributions. To this end, the relevant parameters are defined. These parameters are the optical properties of the UV-C light sources, such as wavelength and emitted optical power, as well as electrical features, like radiant efficiency and consumed power. Furthermore, the characteristics and geometry of the irradiated surfaces as well as the positions of the irradiated surfaces in relation to the UV-C light sources are considered. Because mercury (Hg) lamps are competitive with UV-C light-emitting diodes, a comparative analysis between these two light sources based on the simulation results is also discussed.

## 1 Introduction

The accuracy of the estimation of the germicidal ultraviolet-C (UV-C) dose applied to a surface is a key point for the effective application of UV-C disinfection technology in medical environments, industries, and transportation. The major impact of UV-C disinfection applications is in the medical environment, because ultimately lives can be saved, and the possibilities of disease transmission can be reduced. The transmission of healthcare-associated infections (HAIs) by factors related to patient contact with contaminated surfaces has a higher incidence than previously believed. Surfaces such as furniture, doors, and portable and touch-controlled devices are being now considered as potential sources of nosocomial infections, and, consequently, new methods have been sought for surface disinfection [1–6]. Improper disinfection of rooms and corridors puts patients at risk. Pathogens can remain on surfaces and be transmitted from one person to another through surface contact. Results from a study carried out in 23 hospitals in the United States showed that between 40% and 50% of the surfaces of the patients’ rooms, which must be disinfected by cleaning staff, are not adequately sanitized [7]. The characteristics of a health center, plus the recent incorporation of portable monitoring devices, including mobile phones, mean that there is always the possibility of exchanging microbial agents, as has been demonstrated in outbreaks associated with failures in the sterilization process and disinfection [8]. In the United States, a recent report indicated that diseases associated with HAIs impact patients at an estimated cost of $35 billion [9].

The correct implementation of a modeling technology for UV-C surface disinfection is needed to estimate and define the capability of commercial UV-C devices on the market. A device’s technical characteristics, such as the type of UV-C light source (*e.g.*, a mercury [Hg] lamp or a light-emitting diode [LED]), power consumption, dimensions, geometry of the light sources, and relative positions of the target surface(s), need to be considered in the modeling.

A simple method with a series of procedures is introduced here to estimate the distribution of UV-C radiation on surfaces in order to compute the necessary exposure times to achieve a desired UV-C applied dose. This method seeks to support the design of UV-C devices for these purposes, and to distinguish existing devices from one another based on a device’s capability to disinfect according to its technical characteristics.

We estimated the UV-C irradiance distribution applied to specific surfaces in a medical environment by devices based on Hg lamps and UV-C LEDs. Knowing the estimated values for the irradiance distribution on the surfaces, it is possible to compute the exposure times necessary to achieve the required germicidal UV-C dose.

Our model considers the geometrical properties of superficial plane surfaces (which can be scaled to curved areas) with emphasis on their dimensions and positions with respect to the light source. Properties such as rugosity, moisture, and absorption phenomena can be considered by associating a specific coefficient that quantifies the percentage of irradiance reduction due to the influence of these properties.

**Fig. 1:**
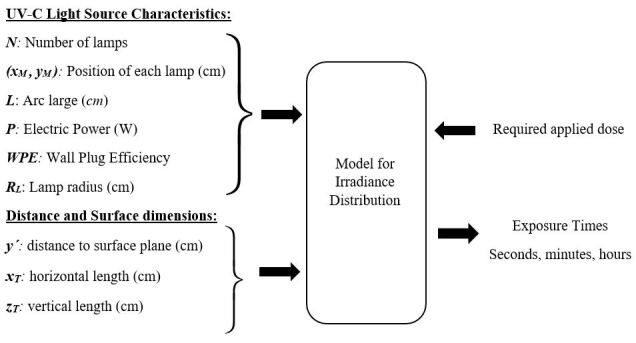
Diagram of the input and output parameters for modeling the irradiance distribution of Hg lamps.

The modeling for Hg lamps can be represented as in the diagram in Fig. 1. The number of lamps and their characteristics, the position of each lamp with respect to its target plane, and the dimensions of target plane positions are the “input values” for the model described in this article.

A similar scheme to Fig. 1 can be used for UV-C LEDs, where the input values are the radiant efficiency “wall plug efficiency” (WPE), the wavelength, the total radiant flux (*Ф*_e_), the number of UV-C LEDs and their positions, the distances to the target surface, and their respective dimensions.

## 2 Photon-Based Units for UV-C Dose and Wall Plug Efficiency

Previous models for applied UV-C dose estimations have been reported for specific water treatment applications [10–12]. These studies were carried out for LEDs and cylindrical Hg lamps. Additionally, standardized protocols have been published for the determination of the UV-C dose response of microorganisms at 253.7 nm, including *in vitro* experiments such as the collimated beam test [13, 14]. Also, various investigations into the use of UV-C LEDs for the estimation of the UV-C dose response of some microorganisms have been published recently [15–18], including the UV-C dose required to inactivate the severe acute respiratory syndrome coronavirus 2 (SARS-CoV-2), the virus that causes coronavirus disease 2019 (COVID-19) [19–22]. As an alternative to the collimated beam test, Bolton *et al*. [23] suggested consideration of photon-based units to estimate the applied UV-C dose. The applied UV-C dose refers to the germicidal energy applied to a surface with a specific wavelength *λ* in the ultraviolet range and differs from fluence used for fluid disinfections (water and air) and the handling of the UV-C dose rate applied to a volumetric flow. The unit for the UV-C dose (*i.e.*, the UV radiant exposure, *H*_e_) is Joules per square meter (J/m^2^), but millijoules per square centimeter (mJ/cm^2^) is commonly used, as in Eq. (1),

$H_{e}(\lambda )=E(\lambda )\Delta t$ (1)

where Δ*t* is the exposure time in seconds, and *E*(*λ*) is the irradiance at specific wavelength *λ*. For the current state-of-the-art UV-C disinfection technologies, Eq. (1) is important because in the last few years new kinds of UV-C light sources have entered the market, such as LEDs that emit monochromatic light at different wavelengths between 265 nm and 285 nm. Table 1 shows values of the UV-C dose required to achieve over 99.9% inactivation of SARS-CoV-2 with two different types of UV-C light sources, Hg lamps and UV-C LEDs, respectively [19, 24–26].

**Table 1:** UV-C dose for COVID-19.

Type	Wavelength (nm)	UV-C Dose (mJ/cm^2^)	Reference
Hg lamp	253.7	3.7	Bianco *et al*. (2020) [24]^a^
UV-C LED	280	37.5	Inagaki *et al*. (2020) [19]

a In addition, Biasin *et al.* [25] reported that a UV-C dose of 3.7 mJ/cm^2^ was sufficient to achieve a more than 3 log_10_ inactivation (99.9%) of the virus at a viral density comparable to that observed in SARS-CoV-2 infection. Also, in a review and analysis of coronavirus photoinactivation studies, Heßling and Vatter [26] reported “the available data [set] reveals large variations, which are apparently not caused by the coronaviruses but by the experimental conditions selected. If these are excluded as far as possible, it appears that coronaviruses are very UV sensitive. The upper limit determined for the log-reduction dose (90% reduction) is approximately 10.6 mJ/cm^2^ (median), while the true value is probably only 3.7 mJ/cm^2^ (median).”

The radiant efficiency, WPE, referred to by the symbol *ƞ*_e_, is defined as the ratio of the total radiant flux *Ф*_e_ to the electrical power *P*_electrical_; see Eq. (2). The value of WPE differs drastically for UV-C LEDs and Hg lamps. The WPE value of UV-C LEDs is less than one twentieth that of Hg lamps. It is important to note that in the case of UV-C LEDs, the WPE value differs for the wavelength of each LED device [15].

$\eta_{e}=\frac{\Phi_{e}(\lambda )}{P_{electrical}}$ (2)

Rattanakul *et al*. [15] listed the *ƞ_e_* values for different types of light sources and wavelengths. Some of the results are summarized here in Table 2.

**Table 2:** Wall plug efficiency (*ƞ_e)_*, kinetic constant of inactivation (*k*), and energy consumption (*E*_3_) as a function of wavelength for a Hg lamp and two UV-C LEDs.

Type	Wavelength (nm)	*η*_e_	*k* (cm^2^/mJ)	*E*_3_ (kW⋅h/m^2^)
Hg lamp	253.7	0.333	8.11	0.009
UV-C LED	265	0.006	8.05	0.41
UV-C LED	280	0.019	5.61	0.17

Table 2 shows the WPE (*η_e_*), the kinetic inactivation constant (*k*) that characterizes the microbiological inactivation rate, and the energy consumption per unit area (*E*_3_). According to the values quoted in Table 2, the WPE of a UV-C LED at 280 nm is three times larger than that at 265 nm. Meanwhile, *k* at 280 nm is only 30% lower than that at 265 nm, and the energy consumption at 280 nm is 50% lower than that at 265 nm. For these reasons, it is more advantageous to work at 280 nm, as shown by Hull *et al.* [27].

### 2.1 Average Dose on a Surface

The average dose applied on a surface can be defined as a product between the average value of the incident irradiance and the time of exposure. An irradiance homogeneity analysis, considering the average irradiance on a surface, has already been proposed by Bolton and Linden, when they defined the “petri factor” [13, 14]. This factor ensures a representative average irradiance value applied on a circular surface, specifically a petri dish, and it is determined by collimated beam experiments. The petri factor is defined as the ratio of the average incidental irradiance of the petri dish area to the irradiance value at the center of the dish. It is used as a corrective factor, providing a greater reading precision of the average irradiance of the surface. According to Bolton and Linden, a well-designed collimated beam apparatus should be able to deliver a petri factor of greater than 0.9. A new parameter, labeled the “homogeneity factor,” can be defined following the criteria used in the “petri factor” definition, that is, with a numerical value representing the accuracy of the average dose applied to a bounded surface. This can then be computed considering the ratio between the average of the incident irradiance value on a specific surface and its maximum value. The “homogeneity zone” can be defined as the bounded surface where the “homogeneity factor” is equal to or greater than 0.9. This homogeneity zone might cover part of the target surface or the entire target area. It is expected that the acceptable homogeneity will be found in some specific areas of the irradiated surface, not inevitably in all analyzed surfaces.

### 3 Modeling of Hg Lamps

**Fig. 2:**
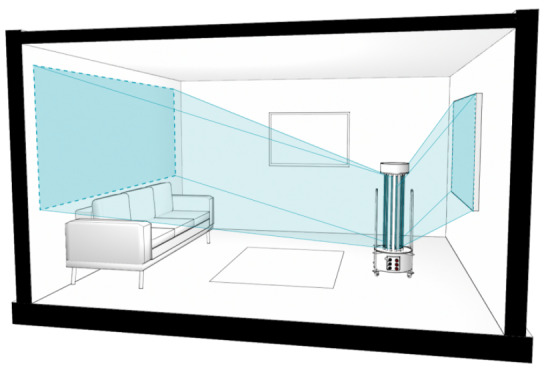
Different surfaces within a room are shown receiving different irradiance distributions applied by the same device. It is important to consider the position of the surface relative to the device.

Kowalski analyzed the problem of UV-C irradiation distribution by single lamps and the irradiation of environments by multiple lamp devices [28]. In this approach, we model the problem for a single lamp and then apply the model to multiple lamps. It is important to consider the target surfaces for the disinfection processes and the target’s relative position with respect to UV-C devices as shown in Fig. 2.

### 3.1 Finite Line Source Model for a Single Lamp

According to Bolton *et al*. [11], the irradiance *E* is defined as the total radiant power from all directions incident to an infinitesimal element of surface area *dS* containing the point under consideration divided by *dS*. The SI unit of irradiance is watts per square meter (W/m^2^). However, milliwatts per square centimeter (mW/cm^2^) is also in common use [11].

**Fig. 3:**
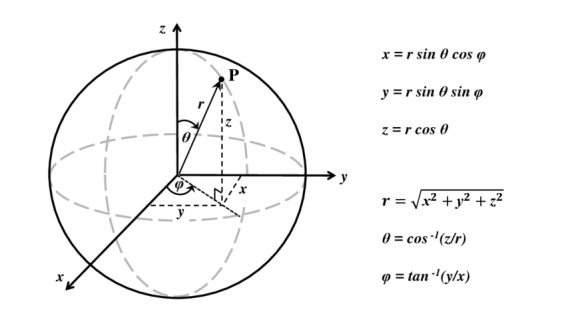
It is possible to determine the irradiance at any point P by applying a homogeneous cylindrical source and using spherical and rectangular coordinates.

**Fig. 4:**
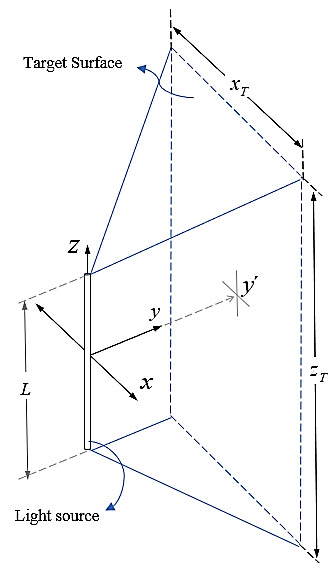

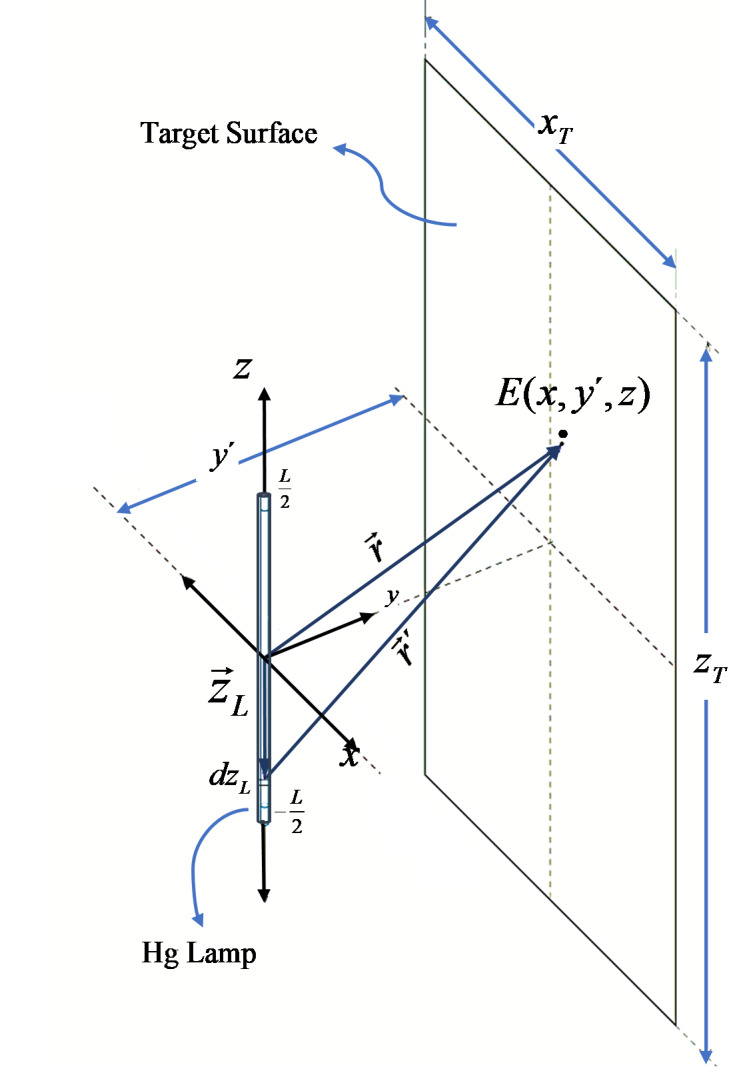
Left: Rectangular coordinates used to define the position in the *x*-*z* plane of the points in the surface that will be used to estimate the value of the irradiance generated by a single lamp. Right: Vector definitions used to compute the rectangular coordinates of any point on the target.

We used rectangular and spherical coordinates to approach the problem mathematically, as shown in Figs. 3 and 4.

When a single lamp is positioned in front of and parallel to a surface, the irradiance of any point on that surface can be determined by using the finite line source model (FLS). This model is based on the premise that a lamp may be approximated by a series of point sources located along the line segment and that the light is emitted spherically from all points on the lamp’s axis [10–12].

For simplicity, we will consider the case of a UV-C light source located on the *z* axis, for which the center coincides with the origin of coordinates of the reference system, and at a *y*' distance from a plane perpendicular to the *y* axis (Fig. 4). If the radius of the lamp is *R_L_*, then *y*' *= y − R_L_.*

In order to find an expression to determine the irradiance or the quantity used to describe the incident optical power per unit of area, for each point of the target plane, vector geometry and coordinate changes are used, moving from spherical to rectangular coordinates (Figs. 3 and 4) as in Eq. (3),

$E\left(\vec{r}\right)=E(r,\theta ,\phi )=E(x,y´,z)$ (3)

The function *E*(*x,y*'*,z*) gives the irradiance at a point in the *x*-*z* plane for a fixed value of *y*'. Its domain can be limited by the dimensions of the rectangular surface to be studied. The target surface has sides represented by *x_T_* and *z_T_* according to Fig. 4. Therefore, the domain of the variable *x* is$-\frac{x_{T}}{2}\leq x\leq \frac{x_{T}}{2}$ and the domain of *z* is bounded according to $-\frac{z_{T}}{2}\leq z\leq \frac{z_{T}}{2}$.

The vector $\vec{r}$ starts from the origin and indicates the position of a point on the target surface. The vector $\overset{\to ´}{r}$ starts from the same position of differential *dz_L_*, as shown on the right-hand side of Fig 4. By geometry, $\overset{\to ´}{r}=\;\;\vec{r}-{\vec{z}}_{L}$ . The vector ${\vec{z}}_{L}$starts at the origin and indicates the *dz_L_* position. Then, it is possible to write the vector $\overset{\to ´}{r}$ in rectangular coordinates as in Eq. (4),

$r´={{\mathrm{(x}}^{2}+y{-}^{2}+{\left(z-z_{L}\right)}^{2})}^{1/2}$ (4)

Irradiance (*E*) is the quantity used to describe the incident optical power per unit of area (W/m^2^). According to Bolton and Keitz [11, 29], it can be defined as the fraction between the total radiant flux (*Ф_e_*) and the area of the sphere that covers the light source, as is shown in Fig. 3 and by Eq. (5),

$E=\frac{\Phi_{e}}{A_{⊗}}$ (5)

This is also true if we consider the differentials of irradiance and radiant flux produced by a differential length of light source *dz_L_*, considering the spherical surface of area related with *dz_L_* as in Eq. (6),

$dE=\frac{d\Phi_{e}}{4\pi r{´}^{2}}$ (6)

The homogeneous cylindrical source is only the arc light of the lamp, the length of which is represented by the variable *L*.

The linear optical power density (LOPD) is defined as the total emitted power divided by the length of the homogeneous linear light source. Keitz calls it “luminous flux per unit length” [29]. Further, we can define the LOPD as the fraction of the power differential *dФ_e_* and length differential *dz_L_* as in Eq. (7),

$\Phi_{L}=\frac{\Phi_{e}}{L}=\frac{d\Phi_{e}}{dz_{L}}$ (7)

Hence, the differential of irradiance caused by a differential of length in the target surface can be written according to Blatchley [30] as in Eq. (8),

$dE=\frac{\left(\Phi_{e}/L\right)}{4\pi r{´}^{2}}dz_{L}$ (8)

The total irradiance produced by the lamp is computed with integration as in Eq. (9),

$E(x,y´,z)=\frac{\left(\Phi_{e}/L\right)}{4\pi }\int_{-L/2}^{L/2}\frac{dz_{L}}{x^{2}+y{´}^{2}+{\left(z-z_{L}\right)}^{2}\;}$ (9)

Applying the integral and considering the lamp dimensions in the *z* axis, we can obtain an expression for the irradiance distribution in the plane as in Eq. (10),

$E(x,y´,z)=\frac{\left(\Phi_{e}/L\right)}{4\pi {\left(x^{2}+y{´}^{2}\right)}^{1/2}}\left[\arctan\left(\frac{\frac{L}{2}-z}{{\left(x^{2}+y{´}^{2}\right)}^{1/2}}\right)+\arctan\left(\frac{\frac{L}{2}+z}{{\left(x^{2}+y{´}^{2}\right)}^{1/2}}\right)\right]$ (10)

This expression is computed with the use of a matrix defined by the values of the irradiance on different points at the target surface [31]. An empirical correction factor can be included in the expression in Eq. (10) related to air absorbance, temperature, and operative conditions, among others. We include a correction factor as suggested by Masschelein related to the decay of irradiance at the borders of an arc light [32] as in Eq. (11).

$E´(x,y´,z)=f_{border}f_{absorbance}f_{operative}E(x,y´,z)$ (11)

### 3.2 Array of Multiple Lamps

If the surface is exposed to multiple lamps, a simple calculation is possible by applying the superposition principle for the electromagnetic radiation. That is, adding the expressions for each individual lamp considering the coordinates of each one of them as in Eq. (12),

$E(x,y´,z)=\sum_{M=1}^{N}E_{M}(x-x_{M},y´-y_{M},z)$ (12)

where (*x_M_, y_M_*) are the coordinates of the center of each *M*th lamp in the plane *x*-*y*. This expression can be extended to the total number of lamps in the systems represented by *N*.

## 4 Modeling UV-C LEDs

We need to find the relevant parameters that can affect the optical output power of UV-C light, and, knowing the total output power, we can then make a better computational simulation to find the optimal distribution of UV-C LED arrays. Various research applications have been developed to study sterilization in bench-scale experiments [17, 18, 33], with a special focus on UV-C irradiance distribution applied to an uncovered glass petri dish containing microbiological samples. For LED sources, the optical output power is a function of the spatial geometry and the operational conditions. The electrical current and temperature of the surface module device (SMD) chip are the most important parameters of UV-C LEDs. While these parameters are not included in the current model, it is important to note that a control system for these parameters in the experimental setup is required to perform a reliable optical characterization [17, 34]. Computations of our model consider that the systems work with thermal handling, allowing LED operations at constant temperatures and with a total control of the system. This ensures a constant value, which is the maximum value specified by the manufacturer.

### 4.1 UV-C LED for Surface Disinfection

The analytical models for studying and optimizing the radiation transfer from a single UV-C LED source to a target make use of simple light distribution. Additionally, an analytical equation of the radiation pattern gives researchers more flexibility in analyzing the light in their applications. Radiometric modeling of a light source can be separated into two classes: near field and far field [35]. In the near-field zone, a source is modeled as an extended area, and it is usually assumed that the distance to the illuminated target is five times shorter than the maximum source dimension. Using the radiometric definition, the far-field model was used in this research. The standardized protocol of UV-C bench-scale disinfection experiments for Hg lamps has been well established by Bolton and Linden using a collimated beam apparatus [8]. Validations of small system devices for water purifications based on UV-C LEDs have also been recently published [27]. Currently, reports on surface disinfection using UV-C LEDs are sparse. In our work, we wanted an analytical expression in rectangular coordinates with which to compute and simulate a UV-C LED array irradiance distribution on a target surface.

### 4.2 Far-Field Modeling for a Single UV-C LED

Various models for UV-C LED irradiance simulations have been published [16, 36, 37], but all of this research is oriented towards water disinfection or petri dish irradiations. We are proposing a simple procedure to obtain the irradiance distribution on a surface caused by a specific UV-C LEDs array. The problem of irradiance distribution applied to a surface has been developed for white LEDs [38, 39]. As the geometry of light distribution is independent of wavelength, it is possible to apply the same mathematical model to describe the UV-C irradiance distribution produced by a single or multiple UV-C LED arrays. However, WPE and wavelength distributions must be considered too.

Most commercial UV-C LEDs are packed in SMD packages and mounted on a metal core printed circuit board. The geometry of SMD UV-C LEDs can be modeled as an imperfect Lambertian source. The light pattern generated by any LED is the result of the sum of three terms: the light directly refracted by the encapsulating lens, the light internally reflected inside the lens, and the light reflected by the reflecting cup. Taking into account all these phenomena, one can assume that the light pattern is a linear combination of certain Lambertian functions and of some other functions generated by diffuse reflections and diffuse refractions, mainly Gaussian and cosine-power angular functions [40]. Therefore, the final radiation pattern should be a linear superposition of these types of functions, which are angularly shifted as a function of the angle of incidence of every traced ray [35, 38, 39].

**Fig. 5:**
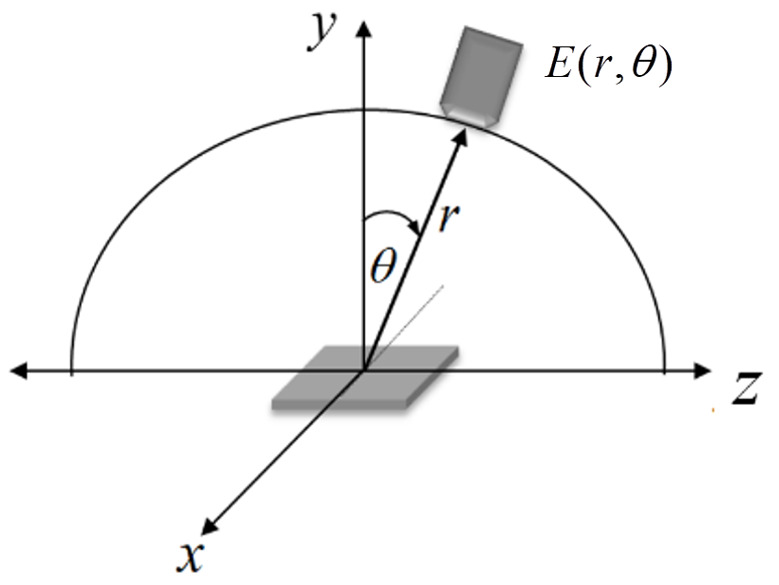
An SMD UV-C LED is shown in the center of the *x*-*z* plane. The detector measures light intensity in polar coordinates for a constant distance from the source.

To numerically model UV-C LED arrays, it is preferable to obtain first the empirical irradiance for a single UV-C LED at a known distance from the target to estimate the irradiance power capability of a specific UV-C LED. The experimental setup can be mounted according to the geometry described in Fig. 5.

UV-C LED irradiance can be modeled using measured radiation profile data. Alternatively, some UV-C LED manufacturers report a relative radiation profile that can be used to obtain values to implement in the simulation models. The total power of the UV-C LEDs reported by the manufacturer can be used in the model [16]. An ideal LED with azimuthal symmetry is a Lambertian emitter; *i.e.*, its luminous intensity distribution is a cosine function of the view angle. In practice, the luminous intensity of a single LED can be treated approximately as an imperfect Lambertian distribution, and, taking into account the reduction of intensity caused by the radial distance, the function of intensity in the target plane is given by Eq. (13),

$E(r,\theta )=c(n)\frac{E_{0}\cos^{m}\theta }{r^{2}}$ (13)

where *E*_0_ is the irradiance value in the direction normal to the surface of the source, *θ* is the polar angle according Fig. 5 (viewing angle), and *c*(*n*) is called the curve-fitting equation. This can be expressed by Eq. (14),

$c(n)=c(0)+c(1)\theta +c(2)\theta^{2}+c(3)\theta^{3}+c(4)\theta^{4}$ (14)

where *c(n)* with *n* = 0.1…4 are the unknown coefficients that are determined by fitting the empirical data [39] as shown in Fig. 5.

The number *m* depends on the relative position of the LED emitting region from the curvature center of the encapsulating device [38, 40]. The number *m* is given by the angular half width value provided by the manufacturer, *θ_1/2_*, also called radiation angle. However, we can obtain an empirical *m* value, defined as the view angle value when irradiance is half the value in the normal direction, (*θ* = 0°). Then, we make measurements to obtain the empirical value of *m* as given by Eq. (15),

$m=-\ln2/\ln\cos(\theta_{1/2})$ (15)

**Fig. 6:**
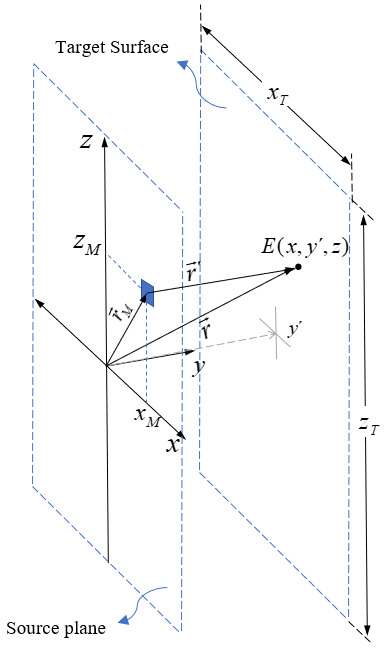
Vector relations used to compute the irradiance at any point on the target surface, where the light source is a single SMD UV-C LED (blue box) placed in the *x*-*z* plane (source plain) with coordinates (*x_M_, z_M_*).

In Eq. (17), we analyze the irradiance distribution over a target plane parallel to the surface of the LED array. The irradiance distribution across the target plane from a single LED in the source plane can be described with spherical and Cartesian coordinates. For the numerical simulation, rectangular coordinates are used. A single LED is located at the position (*x_M_, z_M_*), and the distance between source and target plane is denoted by *y*′ as shown in Fig. 6. The distance r′ of the LED to any point on the target surface can be computed by Eq. (16),

$r´=\left|\vec{r}-{\vec{r}}_{M}\right|={\left({\left(x-x_{M}\right)}^{2}+y{´}^{2}+{\left(z-z_{M}\right)}^{2}\right)}^{1/2}$ (16)

The irradiance distribution at every point on the target plane can be expressed as in Eq. (17),

$E(x,y´,z)=c(n)\frac{\Phi_{e}z^{m}}{{\left({\left(x-x_{M}\right)}^{2}+y{´}^{2}+{\left(z-z_{M}\right)}^{2}\right)}^{(m+2)/2}}$ (17)

where *Ф_e_ = L_LED_A_LED_* according to Wu and Huang [39] in milliwatts (mW), *L_LED_* is the irradiance of the LED in milliwatts per square centimeter (mW/cm^2^) units, and *A_LED_* is the emitting area of the LED in square centimeter (cm^2^). Using Eq. (17) and fitting the distribution of the irradiance data, the irradiance of the LED array composed of multiple UV-C LEDs of the same model can be analyzed.

### 4.3 Modeling Multiple UV-C LED Arrays

**Fig. 7:**
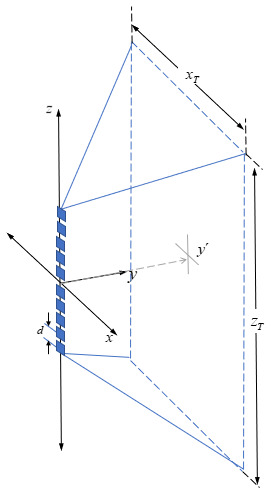
Rectangular coordinates used to define the position in the *x*-*z* plane of the points on the surface that will be used to estimate the value of the irradiance generated by a linear array composed of 10 UV-C LEDs (blue boxes) separated by equal distances at positions on the *z* axis.

As was done in the Hg lamp models previously discussed, a simple calculation is feasible by adding the expressions for each individual UV-C LED and considering the coordinates of each lamp placed on the “source plane” shown in Fig. 7, as illustrated by Eq. (18),

$E(x,y´,z)=\sum_{M=1}^{N}c(n)\frac{\Phi_{e}z^{m}}{{\left({\left(x-x_{M}\right)}^{2}+y{´}^{2}+{\left(z-z_{M}\right)}^{2}\right)}^{(m+2)/2}}$ (18)

where (*x_M_* , *z_M_*) are the coordinates of each *M*th UV-C LED in the plane *z*-*x*. This expression can be extended to the total number of lamps in an LED array. This configuration can be compared with straight cylindrical Hg lamps.

## 5 Results

This section shows the application of the models proposed for the two principal UV-C light sources currently used, Hg lamps and UV-C LEDs. It is possible to obtain a matrix in which the elements are the intensity values of specific points in the irradiated target area with the previously described Cartesian coordinates. The values of the matrix can be statistically and graphically analyzed. The matrix dimensions will be determined according to the accuracy of simulations and dimensions of the target surface.

For Hg lamps and UV-C LEDs, the irradiance distribution on a surface can be studied considering the homogeneity of the light distribution. A similar problem has been studied for collimated beam experiments to obtain the UV-C dose-response curve of specific microorganisms. The collimated beam experiments with Hg lamps or UV-C LEDs were designed to irradiate the circular surface of an uncovered petri dish.

In our case, we are applying the irradiance distribution on a surface, but for other surface sizes. Considering the study of the homogeneity on these surfaces, we use the term “homogeneity factor.” In standard surface disinfection applications, the target surface dimensions are considerably larger than those of a petri dish.

### 5.1 Hg Lamps

The simulations were computed considering four specific Hg lamps readily available on the market, and considering their features as specified by the manufacturers. The specific lamp characteristics are summarized in Table 3.

**Table 3:** Characteristics of Hg lamps.^a^

Specimen	Lamp Model	Arc Length (mm)	Electrical Power (W)	UV-C Output Power at 254 nm (W)	WPE
Lamp 1	GPH436T5L4PSE	360	48	13	0.27
Lamp 2	GHO36T5L4PSE	755	87	28	0.32
Lamp 3	GPH893T5L4PSE	815	95	30	0.32
Lamp 4	GHO64T5L4PSE	1421	155	54	0.35

^a^ Values obtained from catalogs available on the market provided by manufacturers [41, 42].

To accomplish a comparative study between these four lamps, we performed simulations to estimate irradiance values in the target plane placed symmetrically in front of the lamps, where the center is the origin of coordinates according to Fig. 4. We specifically considered a target area 0.5 m wide and 1 m long, which was represented as −25 ≤ *x_T_* ≤ 25 (cm) and −50 ≤ z*_T_* ≤ 50 (cm).

The position of the lamp, the arc length, its diameter (they all had the same diameter equal to 5/8 inch, equivalent to 15 mm), the electric power, its WPE, the distance to the target surface, and the required dose are input variables to be considered in our model according to Fig. 1. The output variables report the spatial distribution of irradiance on the target area. With the output values, statistical techniques can be applied and comparisons among averages, minima, and maxima can be studied to estimate the appropriate implementation of a surface disinfection process using that UV-C radiation source.

#### 5.1.1 Distance Dependence of the Irradiance Distribution on a Target Surface

**Fig. 8:**
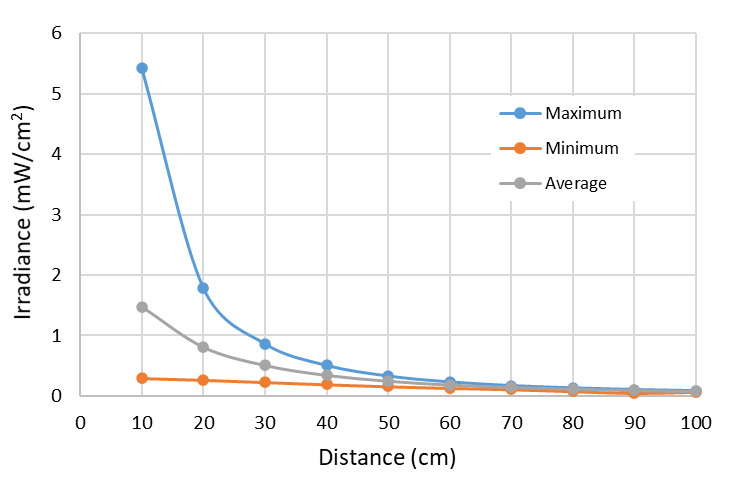
Irradiance variation depending of the distance for maximum, minimum, and average values for lamp 1. Lamp 1 is described in Table 3.

The relation between the irradiance and distance is known, and hence, using the data obtained by simulations applying our technique, it is possible to analyze the relation between target surface distance and the minimal, maximal, and average values of irradiance on the target surface. Additionally, it is possible to show the irradiance distribution values graphically, with the input values previously known, as is shown in Fig. 8.

The differences among the maximum, minimum, and average values as a function of distance from the target surface decrease for lamp 1 (Fig. 8). The average values are representative of conditions when the distance exceeds a certain value, which allows us to define our “far-field zone.”

**Fig. 9:**
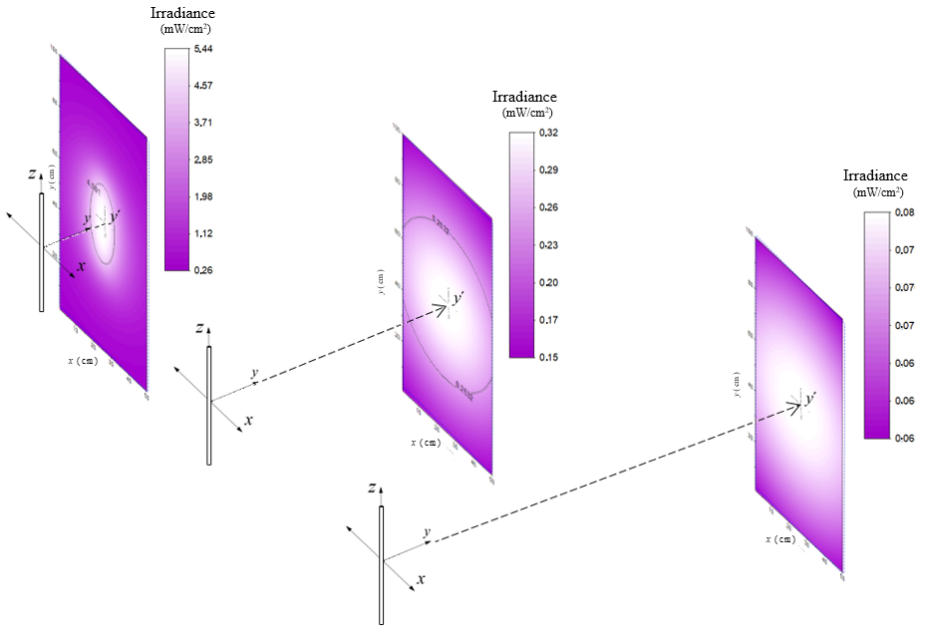
Irradiance distribution on the target surface for Hg lamp 1 at 10 cm, 50 cm, and 100 cm, respectively. The black line at 10 cm and 50 cm selects the “homogeneity zone,” respectively.

The values obtained in the mentioned matrix can be represented graphically using a color scale as is depicted in Fig. 9 for lamp 1 for three different cases: at 10 cm, 50 cm, and 100 cm distance. It becomes possible to discern the spatial irradiance distribution in the plane, and therefore to analyze maxima and minima according to the selected configuration.

In the first two graphs of Fig. 9, the black line selects the “homogeneity zone” for distances of 10 cm to 50 cm, respectively. Increasing the distance increases the area of the homogeneity zone, implying that the light distribution is more homogeneous at larger distances. For example, choosing 100 cm, the results show that the homogeneity in the rectangular area (50 cm wide and 100 cm high) is superior to 0.9, and therefore at 100 cm, it is possible to consider the average irradiance over the entire surface as a dependable value. Nevertheless, the larger distance yielding a better homogeneity also implies that the irradiance decreases with the square of the distance.

**Fig. 10:**
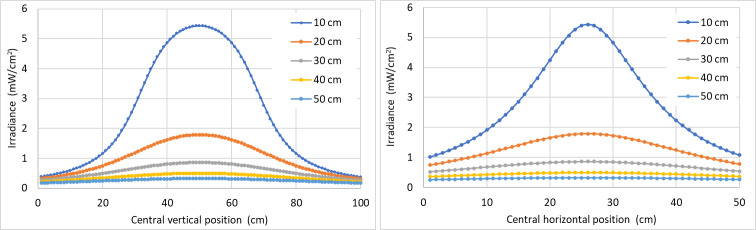
Irradiance distribution of central lines in vertical and horizontal directions, applied on a target plane for lamp 1 at 10 cm, 20 cm, 30 cm, 40 cm, and 50 cm. Lamp 1 is described in Table 3.

It also is possible to plot vertical and horizontal graphs of the irradiance through the center of the target area (Fig. 10).

#### 5.1.2 Average Irradiance and Exposure Times Required for a Specific UV-C Dose

Irradiance isolines can be defined as the lines formed by the points on the target surface with equal value of incident irradiance. These lines define the outline of areas of interest where the homogeneity is statistically analyzed. It is feasible to compute the factor of homogeneity defined in Sec. 2.1. for all the inner points of each isoline (or a group of these) and then establish the irradiance value of the isoline that borders the specific area where the factor of homogeneity is equal to or greater than 0.9. This specific isoline delimits the area where the homogeneity of the incidental irradiance is useful. This zone will be called “homogeneity zone.”

**Fig. 11:**
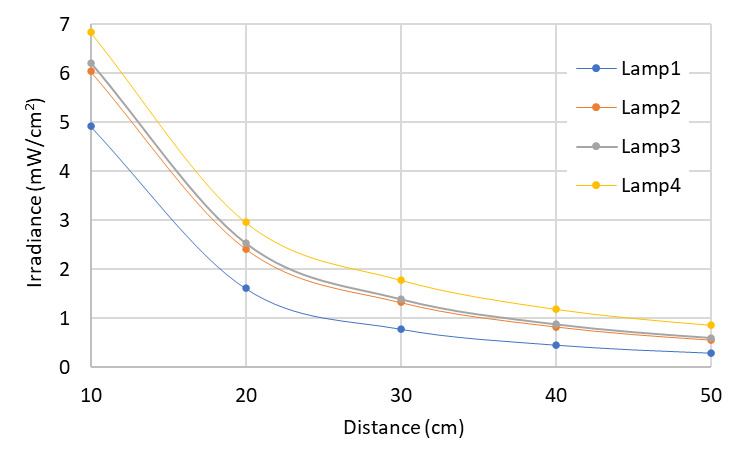

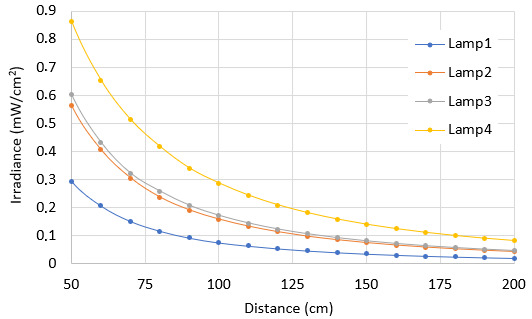
Average irradiance distribution and its distance dependence for four different lamps. Lamps are described in Table 3.

A criterion that defines the reliability of the germicidal action has been established. Suppose that a specific germicidal dose must be applied with a Hg lamp emitting at a wavelength of 253.7 nm onto a surface, *e.g.*, a known dose for SARS-CoV-2 of 3.7 mJ/cm^2^, to obtain inactivation of 99.9% of the initial concentration of microorganism, according to Table 2. It is then possible to determine the required exposure times Δ*t* according to distance and type of lamp using our model. Considering the characteristics of the lamps described in Table 3, it is feasible to compare the variation of the average irradiance with the respective “homogeneity zone” for the distances to the target surface for each lamp separately. This can be observed in Fig. 11. In addition, it is possible to estimate the germicidal performance of each lamp according to its characteristics, such as size, power, and position with respect to the target area to be treated.

**Fig. 12:**
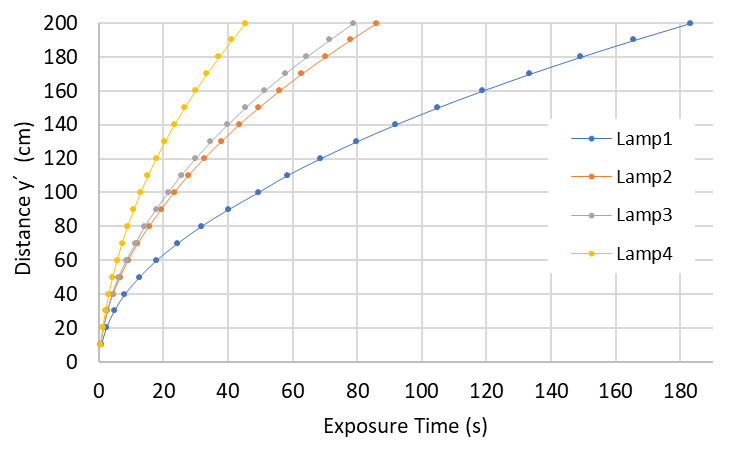
Dependence of distance and exposure time required to achieve a set value of UV-C dose. Lamps are described in Table 3.

Let us assume that a SARS-CoV-2 dose of 3.7 mJ/cm^2^ must be applied to a surface. Using Eq. (1) and the average irradiance values in the surface area, we can determine the required exposure times for different lamp types and different distances as shown in Fig. 12.

As seen in Fig. 12, lamps with higher power require lower exposure times. As distance increases from the UV-C source, exposure times increase accordingly. It is easy to conclude that a reduction in exposure times can be obtained by increasing the power of the UV-C sources or decreasing the distance between the lamps and the target surface. Decreasing exposure times can be achieved as well by including more lamps in the process, known as multilamp systems, or using more than one multilamp device.

### 5.2 UV-C LEDs

Three diverse LED devices available on the market were modeled, taking into account their features as specified by the manufacturers. The specific characteristics collected from the data sheets provided by the manufacturers considering the maximal operation rates for the forward current are shown in Table 4.

**Table 4:** Characteristics of UV-C LEDs.^a^

Specimen	Company Name	Model	Wavelength (nm)	Radiant Flux (mW)	Forward Current (mA)	Radiant Angle *ϴ*_1/2_
LED 1	Nikkiso	VPS164	280	40	350	60
LED 2	Luminus	XST-3535-UV-A60-CE280	280	100	650	30
LED 3	Violumas	VC2X2C48L6-275	275	192	1400	30

^a^ Values obtained from catalogs available on the market provided by manufacturers.

To accomplish a comparative study between these three types of UV-C LEDs and the Hg lamps (Table 3), we performed simulations to estimate irradiance values in the target plane placed symmetrically in front of a linear LED array composed of 10 UV-C LEDs, as shown in Fig. 7. A linear set of 10 UV-C LEDs was placed in a pattern similar to that of the Hg lamps discussed previously in order to achieve a comparative assessment. In this configuration, we observed that the edges of the irradiance pattern were virtually zero, implying that no statistical analysis was meaningful. Not seeking to evaluate averages in nonhomogeneous areas, we evaluated the data in smaller surfaces when irradiating with UV-C LEDs. We specifically considered a target area 50 cm wide and 1 m long, which was represented by −25 ≤ *x_T_* ≤ 25 (cm) and −50 ≤ z*_T_* ≤ 50 (cm), according to Fig. 7.

#### 5.2.1 Distance Dependence of the Irradiance Distribution on a Target Surface

**Fig. 13:**
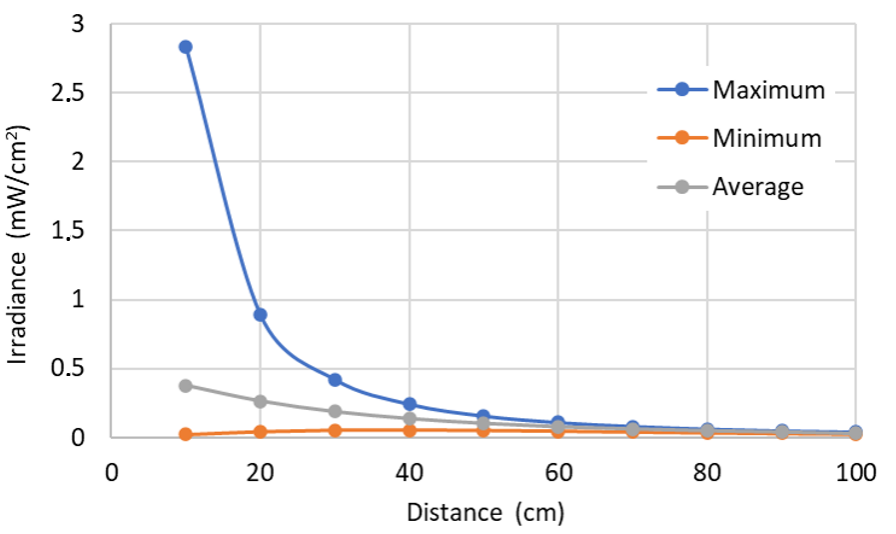
Irradiance variation depending on the distance for maximum, minimum, and average values for the array with LED 1. LED 1 is described in Table 4.

As with the Hg lamps, it is possible to compare average values, and maxima and minima values for an array of 10 UV-C LEDs. Figure 13 shows the case of an array assembled by LED 1 according to the description in Table 4. Using data obtained by simulations that apply our technique, it is possible to analyze the relation between target surface distance and the maximum, minimum, and average irradiance values on the target surface (Fig. 13). For LED 1, these values differ considerably, as shown in Fig. 13.

**Fig. 14:**
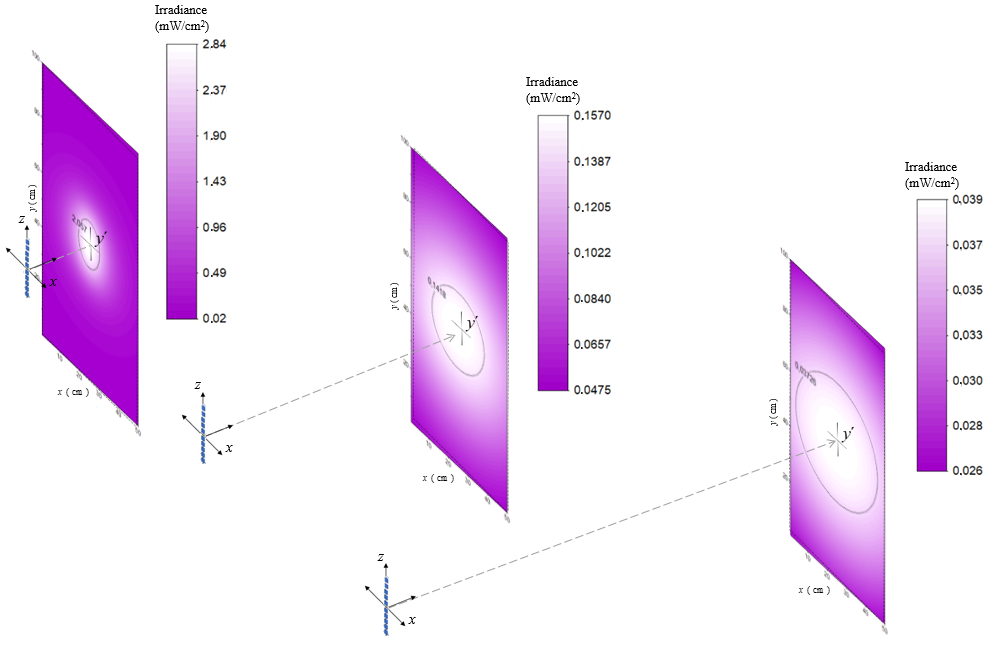
Irradiance distribution on the target surface irradiated by the array composed of LED 1 light sources at 10 cm, 50 cm, and 100 cm, respectively. The black line selects the “homogeneity zone,” respectively.

As previously mentioned, it is possible to represent the values of the matrix graphically using a color scale, as shown in Fig. 14. This can be performed with an array of multiple LED 1 light sources (described in Table 4). Three different cases are shown corresponding to irradiance patterns at distances of 10 cm, 50 cm, and finally 100 cm away of the target surface.

**Fig. 15:**
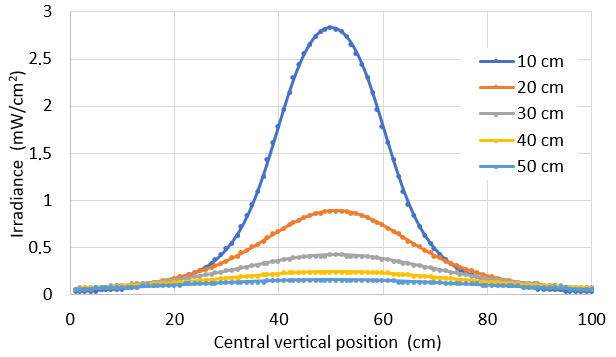

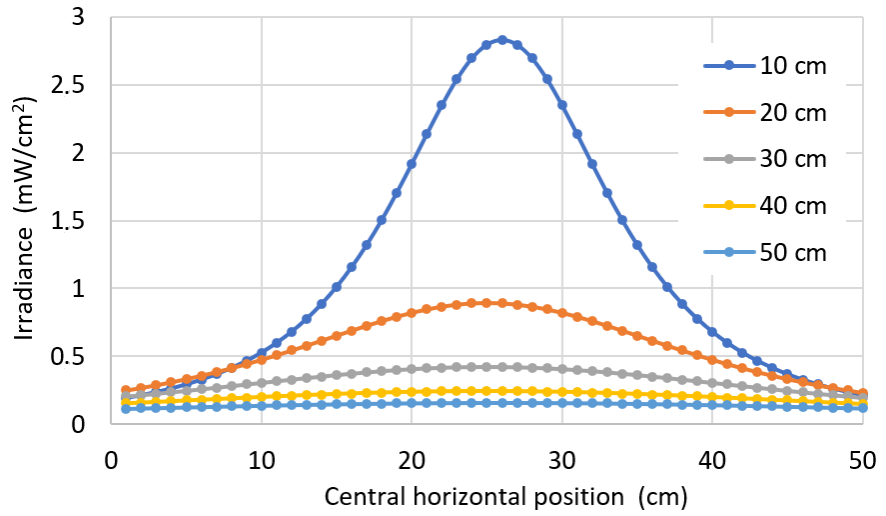
Irradiance distribution on the target surface for LED 1 at 10 cm, 20 cm, 30 cm, 40 cm, and 50 cm. LED 1 is described in Table 4.

We also plot the variation of irradiance for LED 1 according to vertical and horizontal lines across the center of the target surface (Fig. 15).

It is interesting to notice from Fig. 15 that some points close to the borders display an increased irradiance generated by a source 20 cm apart (orange curve) as compared to the one at 10 cm (blue curve). This can be interpreted as the effect of “radiation angle” of the LEDs, displayed on Table 4.

#### 5.2.2 Average Irradiance and Exposure Times Required for a Specific UV-C Dose

**Fig. 16:**
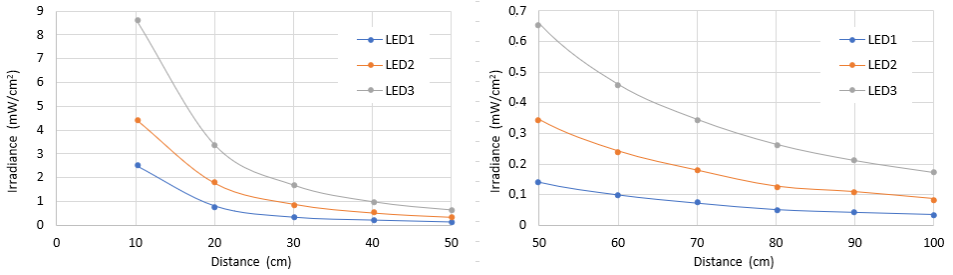
Average irradiance and its distance dependence for three different LEDs arrays. LEDs are described in Table 4.

As with the Hg lamps, we applied the model to the UV-C dose value required for a 99.9% inactivation rate of SARS-CoV-2 with a UV-C LED emitting at a wavelength of 280 nm, given a value of 37.5 mJ/cm^2^ [19]. With this dose value, it is possible to determine the exposure times at different distances. Considering the characteristics of the LEDs described on Table 4, it is feasible to compare the variation of the average irradiance of the respective homogeneity zone with the distance to the surface for each LED array separately, as is shown in Fig. 16.

**Fig. 17:**
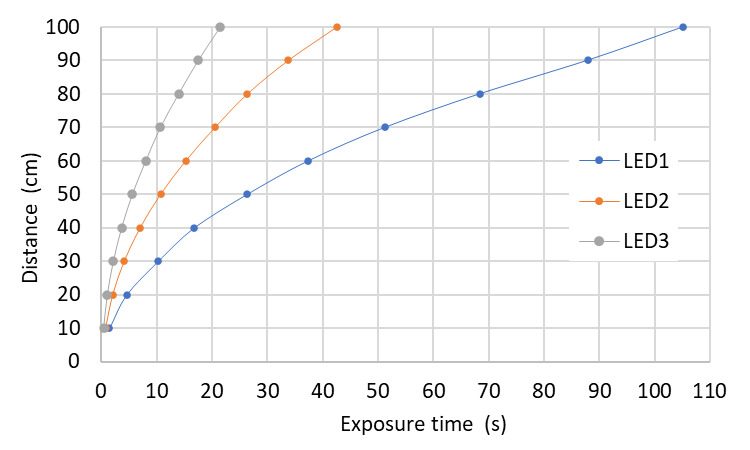
Dependence of distance and time required to achieve a set value of UV-C dose with three different LED arrays. LEDs are described in Table 4.

Similar to results obtained with the Hg lamp simulations, it can be seen that UV-C LEDs with higher power require lower exposure times at longer distances (Fig. 17).

#### 5.2.3 Spatial Distribution and “Radiation Angle ϴ_1/2_” of the UV-C LEDs

**Fig. 18:**
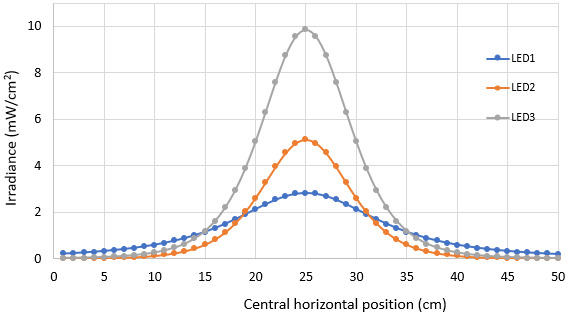
Irradiance distribution on the target surface for the LED arrays with 10 LEDs made up by LED 1, LED 2, and LED 3 types, respectively, at the same distance of 10 cm from the target surface. LEDs are described in Table 4.

The homogeneity of irradiance distribution on the surface can be studied by comparing average, maxima, and minima values as displayed in Fig. 13. For UV-C LEDs, these calculations depend on the “radiation angle” defined in Eq. (15). The reported radiation angles for the three LEDs in this study are listed in Table 4. Conventionally, one uses the average irradiance value to estimate the germicidal dose applied to a surface. Before showing the obtained results with respect to the variation of the average irradiance as a function of the distance to the target plane, we show the variation of the irradiance in the horizontal axis (*x* axis) of the target surface. Figure 18 shows the simulation results for the three different arrays made up by LED 1, LED 2, and LED 3 types, respectively, at a constant distance of 10 cm from the target surface.

Notice that the irradiance generated by the LED 1 (blue curve) device at the edges of the graph exceeds that of LED 2 and LED 3 because of its radiant angle *ϴ_1/2_* = 60°, despite the higher radiant power of the LED 2 and LED 3 types. Both LED 2 and LED 3 types have radiant angles of only 30°, demonstrating the importance of the radiant angle for homogeneous distributions.

### 5.3 Comparison of Irradiance Between Hg Lamps and UV-C LEDs

The WPE is up to 20 times larger in Hg lamps than in LEDs [43]. We compared the performance of the Hg lamp 1 with the array of 10 UV-C LEDs composed of the LED 3 type, which are described in Tables 3 and 4.

**Fig. 19:**
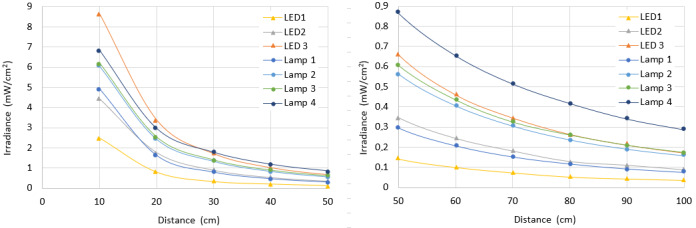
Average irradiance depending on the distance for Hg lamp 1 and LED 3.

Simulation results (Fig. 19) show the average irradiance in the homogeneity zone as a function of the distance for a specific surface, considering variable distances, computed for a target surface 50 cm wide and 100 cm high. The distances range from 10 cm to 100 cm between the source and the target plane.

#### 5.3.1 Comparison of Average Irradiance Distributions

**Fig. 20:**
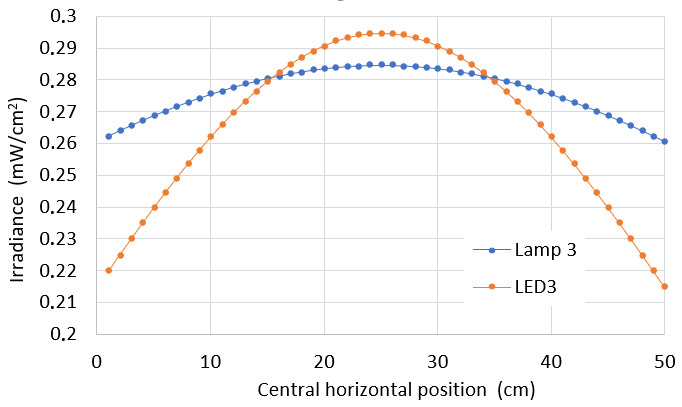
Irradiance depending on the distance for comparative analysis between Hg lamp 1 and LED 3.

In this simulation, results from Hg lamp 3 and the LED 3 array at 80 cm distance from the target surface were compared to clarify the differences in both irradiance distributions yielding the same average irradiance values. The best approach to appreciate the irradiance behavior on a surface is to generate graphs of the intensity vertically and horizontally through the target plane as shown above. A comparison of the irradiance variation can be observed in Fig. 20, displaying a central horizontal line.

**Fig. 21:**
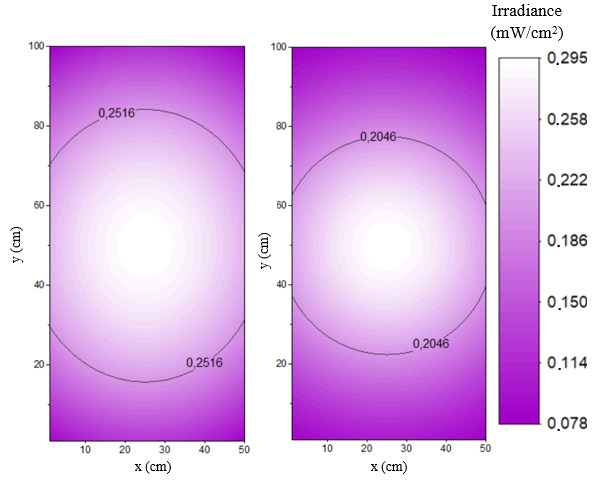
Irradiance distribution on the target surface irradiated by Hg lamp1 and an LED array composed of LED 3 at 40 cm.

Our model allows a visual comparison and a statistical evaluation of the irradiance intensity distribution on rectangular surfaces with dimensions and distances well established in the cases of Hg lamps and UV-C LEDs as in shown in Fig. 21. All parameters discussed previously have to be considered to characterize the germicidal capacity of a system. In addition, one has to remember that a given average does not represent the same intensity distribution with the same maximum and minimum values on the same surface.

## 6 Conclusions

The calculations based on the mathematical models described in this paper and the results obtained with computational simulations are versatile for multiple potential applications. All equations and mathematical considerations have been taken from previous studies, some of which focused on common light sources and others specifically on UV-C disinfection technologies [16, 30, 31]. The presented procedures can be applied to single or multiple UV-C light source configurations of Hg lamps and LED arrays. Various spatial distributions of target surfaces can be assessed.

This work is a starting point for future computable models of increasing predictive capacity. For this, it is important to develop experimental setups in order to collect measurements to be fitted with the irradiance distributions. Data obtained with the procedures presented in this article may be combined with experimental data to contribute to or refine standardized measurements and to verify these observations. These clear additional steps are currently under progress.

Our model allows estimation of the optimal configurations for the design of new devices. Additionally, it permits an estimate of the germicidal capacity of any device based on Hg lamps or LEDs, considering its characteristic parameters as identified in this study. Despite the lack of empirical measurements with which to validate the proposed model, we believe it will yield satisfactory results if the correction parameters are obtained in an appropriate way based on calibrated optical measurements. Both models, operating with Hg lamps and UV-C LEDs, are based on previous published studies, where the models have been compared with the measurements with very satisfactory results.

The procedures described in this paper permit the collection of simulated irradiance distributions for each configuration of interest. Using these simulation data, statistical analysis can be realized to help develop effective protocols for disinfection of any surface, especially in medical environments. Additionally, graphic representations can be made to illustrate what happens at the respective surface when it is irradiated, providing input to support and control the disinfection process with UV-C radiation sources.
